# THE CEMENTED DOUBLE MOBILITY CUP IN HIP REVISION: DIFFERENT POSSIBILITIES OF USE

**DOI:** 10.1590/1413-785220233101e256913

**Published:** 2023-04-17

**Authors:** Jaime José Morales de Cano, Estela Trias

**Affiliations:** 1 Hospital Universitario de Vic, Vic, Vic, Spain.

**Keywords:** Reoperation, Procedures, Operative Surgical, Hip Replacement, Total, Follow-Up Studies, Reoperação, Procedimentos Cirúrgicos Operatórios, Artroplastia Total do Quadril, Seguimentos

## Abstract

**Introduction:**

The aim of our work is to review those patients who underwent prosthetic hip revision surgery in our hospital considered to be patients at high risk of dislocation or recurrent dislocation, and who underwent a double mobility cemented cup (CMD). Analyzing the different ways to place these cups and the clinical results and reluxations.

**Material and methods:**

The 69 cases comprised 34 men and 35 women with a mean age of 77,39 years. The mean follow-up was 4.7536 years. The type of intervention performed varied according to the cause of the intervention, the acetabular bone stock and the state of the primary cup. In the cases in which there was a good fixation of the primary metalback, we opted to carry out a cementation of the cemented DMC into the existing well-fixed metal acetabular shell, this occurred in 23 cases. In the cases where there was loosening of the primary cup but there was a good bone stock, a CMD was cemented into the bone (21 cases). In the cases where there was a Paprosky type III we cemented a DMC to a Bursch-Schneider reinforcement ring together with the placement of a cancellous bone graft (25 cases).

**Results:**

The clinical evaluation at the end of the follow-up, according to the MD Scale, showed the mean value was 16.454 (SD 0.79472), with a survival at the end of the follow-up of 100% of the placed DMC.

**Conclusion:**

The use of cemented DMC is a good solution in the replacement of THA, especially in cases of reluxation or risk of dislocation due to personal or technical predisposing factors. The use of these DMC cemented can be directly to the bone, into the existing well-fixed metal Shell, or cemented to a reinforcing ring, depending on the acetabular defect. ***Evidence Level III; Comparative Case Series***.

## RESUMO

**Introdução:**

Revisar os pacientes que foram submetidos à cirurgia de revisão protética de quadril neste hospital, considerados como pacientes com alto risco de luxação ou luxação recorrente, submetidos a cirurgia por acetábulo cimentado de dupla mobilidade (CMD). Analisando as diferentes formas de posicionamento desses copos, seus resultados clínicos e reluxações.

**Material e métodos:**

Os 69 casos correspondiam a 34 homens e 35 mulheres com uma idade média de 77,39 anos. O tempo médio de acompanhamento foi de 4,7536 anos. O tipo de intervenção realizada variou de acordo com a causa da intervenção, o estoque ósseo acetabular e o estado do copo primário. Nos casos em que houve uma boa fixação do metal primário, optouse por realizar uma cimentação do DMC cimentado na cúpula acetabular metálica firme existente, o que ocorreu em 23 casos. Nos casos em que houve um afrouxamento acetabular primário com um bom estoque ósseo disponível, cimentou-se um CMD (21 caixas). Nos casos em que havia um Paprosky tipo III, cimentou-se um DMC a um anel de reforço Bursch-Schneider juntamente com a colocação de um enxerto ósseo esponjoso (25 caixas).

**Resultados:**

A avaliação clínica realizada no final do acompanhamento, de acordo com a Escala MD, mostrou que o valor médio foi de 16,454 (DP 0,79472), com uma sobrevivência ao final do acompanhamento de 100% do DMC inserido.

**Conclusão:**

O uso do DMC cimentado pode ser uma boa solução para substituição do THA, especialmente em casos de reluxação ou risco de deslo-camento devido a fatores de predisposição pessoais ou técnicos. O uso destes DMC cimentados pode ser realizado diretamente ao osso, dentro da cúpula metálica fixa existente, ou cimentados a um anel de reforço, dependendo do defeito acetabular. ***Nível de Evidência III; Série de Casos Comparativos***.

## INTRODUCTION

One of the most important complications in prosthetic hip surgery is dislocation. According to Woolson,^1^ the incidence found in their series reaches 3.5% of the 10,500 cases of primary total hip replacement (THA) that they collect. But this incidence is much higher in revision surgery. Grigoris finds up to 25% of dislocations in the review.^2^ According to the 2019 US THA registry, in 2017 the main reason for revision was due to prosthetic instability and in 2018 it was the second after infection.^3^

Rowan recently conducted a literary search to assess historical perspectives and current strategies to prevent dislocation after primary THA. This study included 3,458 articles and included 154 in its analysis.^4^ There are two groups of causes that can favor prosthetic instability and therefore dislocation: patient-specific causes and technical causes. Among the patient's own causes, it has been shown that age, body mass index above 30Kg / m2, lumbosacral pathology, rheumatoid arthritis, muscle atrophy, history of interventions on the same hip, can be factors favoring instability. Among the technical causes, the approach route, the size of the femoral heads, the anteversion of the cotyloid component, the inclination of the acetabular component, the relaxation of the soft tissues, the femoral retroversion. Taking into account all the favorable effects of prosthetic instability, preoperative planning is very important in a primary THA, but much more in revision surgery. It is also important to be able to do a dynamic test with the trial prosthetic components during surgery, such as the push-pull test, to assess the soft tissue tension and the stability of the prosthesis. But not all approaches facilitate it in the same way.^4, 5^

In these circumstances, any technical help that can improve the stability of the surgery and especially in revision surgery, is welcome. In revision surgery, patients already have a history of surgeries with poorer quality soft tissue and many times with bone defects that are difficult to resolve. There is no doubt that double mobility cups (DMC) have become an aid to improve stability. Good results have been reported in the use of DMC in primary surgery,^6,7^ also in patients with neuromuscular problems^8-13^ and even in revision surgery,^14-16^ but there are still no conclusive data on the use of DMC in revision surgery. in patients with a neurological history, or older patients. The aim of our work is to review those patients who underwent prosthetic hip revision surgery in our hospital considered to be patients at high risk of dislocation or recurrent dislocation, and who underwent a cemented CMD. Analyzing the different ways to place these cups and the clinical results and reluxations.

## MATERIAL AND METHODS

In total, 69 patients underwent was operated between January 2010 and December 2001, placing an Avantage® DM cemented Shell (Zimmer Biomet, Warsaw, USA). the study was conducted in line with the established ethical guidelines of the hospital: each patient at the hospital was asked to sign an informed consent whether to let his or her data public or private for future access, and only open access medical records were reviewed by the authors of the study. Since this is an observational retrospective study, it does not describe experimental studies on either humans or animals and so it does not need any ethical approval.

The external surface of the cemented Avantage Reload metal shell has a bright polish (Ra max 0.4 µm), and the inner articulate surface is highly polished. In all cases, a cobalt-chrome femoral head was used. The diameter of the heads depended on the size of DMC used. Highly cross-linked polyethylene liner infused with vitamin E (GUR 1050) was used on all cups.

All patients were operated on by 2 highly experienced orthopedic hip surgeons. Cefazolin 2gr was administered intravenously before surgery and twice after the operation with an interval of 8 hours. The patients underwent antero-external or posterolateral surgery and received the same rehabilitation program, which allowed full loading immediately after surgery. Thromboembolic prophylaxis with low molecular weight heparin was performed, and blood saving protocol with tranexamic acid. In the postoperative period, surgical bleeding and days of hospital admission were analyzed. The indications for these implants in particular were: patients without age limits who require revision surgery due to implant instability, or revision surgery with their own or technical risk factors for prosthetic instability. All cases were submitted to preoperative planning.

**Figure 1:**
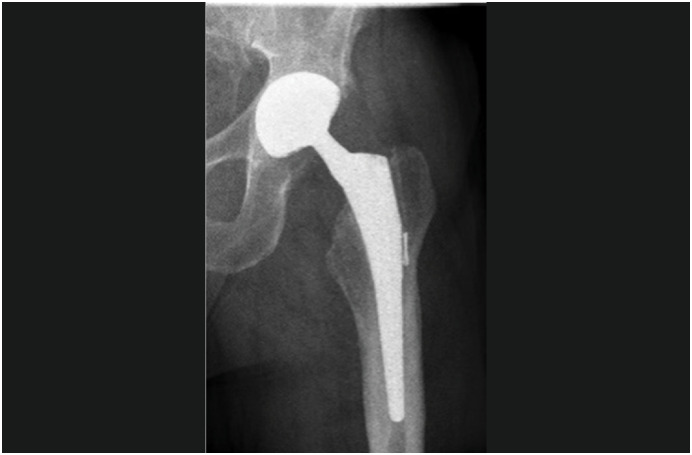
The case of an 86-year-old woman operated on for right PTC infection, with replacement of both prosthetic components. Placement of cemented DMC to the bone.

**Figure 2:**
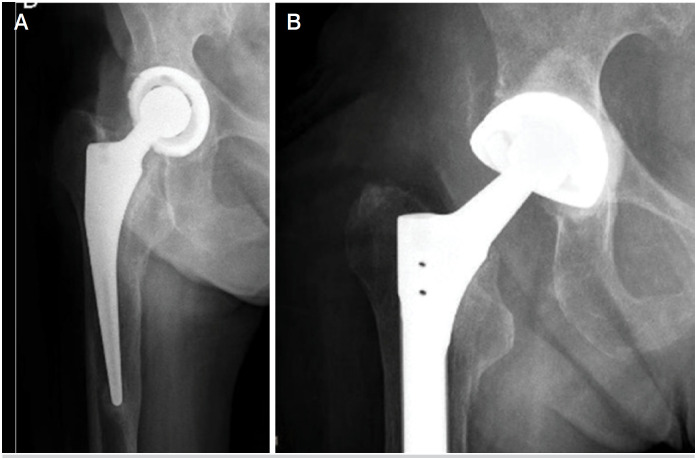
The case of a 79-year-old man with septic loosening of total hip arthroplasty (A). Placement of cemented DMC into DMC into the existing well-fixed metal shell (B).

**Figure 3:**
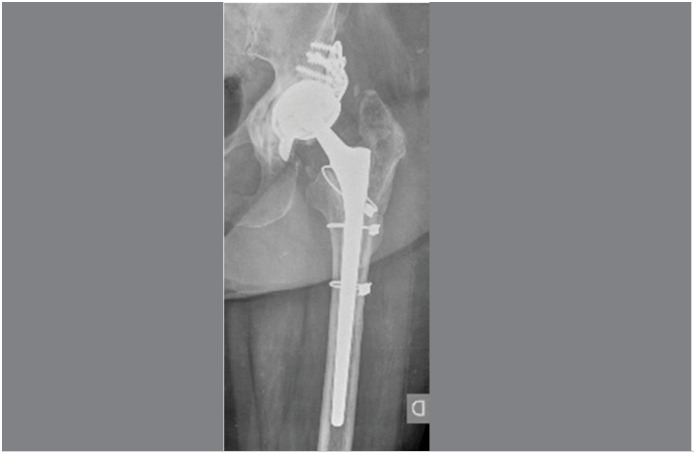
The case of an 82-year-old woman with aseptic acetabular loosening. Placement of cemented DMC into the Bursch-Schneider ring.

The 69 cases comprised 34 men and 35 women with a mean age of 77,39 years (range between 46 and 89 years) at the time of surgery. The mean follow-up was 4.7536 years (SD 2.075) between 3-16 years. The mean time elapsed between primary surgery and revision was 12.79 years (SD 6.7814) between 1–28 years. The mean body mass index was 27.40 kg / m2 (range 17.38 to 43.40). The distribution of patients according to diagnosis was: in 23 cases a recurrent prosthetic instability, in 35 cases a prosthetic loosening with risk of instability, in 7 cases they were due to prosthetic replacement caused by a Vancouver type B or C periprosthetic fracture, and in 4 cases septic exchange with risk of dislocation. (Table 1) The type of intervention performed varied according to the cause of the intervention, the acetabular bone stock and the state of the primary cup. In the cases in which there was a good fixation of the primary metalback, we opted to carry out a cementation of the cemented DMC into the existing well-fixed metal Shell (Figure 1), this occurred in 23 cases. In the cases where there was loosening of the primary cup but there was a good bone stock, a DMC was cemented into the bone, this happened in 21 cases. (Figure 2) And in cases where there was a Paprosky type III A or B bone defect, we cemented a DMC to a Bursch-Schneider reinforcement ring together with placement of a cancellous bone graft in 25 cases. (Figure 3) Patients were clinically assessed using the Merle d’Aubigné (MD) score preoperatively and at the end of follow-up. The mean preoperative assessment was 6.9276 (SD 2.068669). The radiological evaluation was carried out by means of a standard anteroposterior radiography of the pelvis and lateral hip, verifying the migration, osteolysis and signs of radiolucency, as well as the position of the cup and the position of the femoral stem.

**Table 1:** Description of the re-revised study population.

		Statistical significance
Age, years, mean (range)	77, 39 (46-89) SD 9.4217	
Sex	Female 35, male 34	P>0.005
Side,Right/Left	R 42, L 27	
Body mass index, kg/m^2^,mean (range)	27,40 kg / m2 (range de 17,38 a 43,40)	
Operations Previus	2,3 (1-4) SD 6,7814	
Years since the first Operation	12,79 (1-28)	
Preoperative MD	6.9276 (SD 2.068669)	
Cause Surgery	-Loosening 35 cases -Dislocation recurrent 23 cases -Fractura periprotesica 7 cases -Infection 4 cases	P>0.005
Type Surgery	-Anillo Bursch-Schneider + ingerto + Cemented DMC 25 cases -Cemented DMC in metalback 23 cases -Cemented DMC in boone 21cases	P>0.005
Follow-up	4,7536 years (SD 2.075)	
MD postop	16.454 (SD 0,79472)	

Descriptive data are presented as median and range or by mean and standard deviation. The level of significance for all the analyzes performed was established at p ≤0.05. Statistical analyzes were performed using SPSS 26.0 (SPP Inc., Chicago, IL, USA).

## RESULTS

We had no intraoperative complications. In the postoperative period, a mean bleeding in the drainage of 275cc (between 225-700cc) was registered. Only 6 cases (8.69%) required transfusion. The mean hospital stay was 6.5 days (range 5-7 days). The patients began sitting 24 hours after the intervention and walking with partial load between 24-48 hours after the intervention. In the moment of hospital discharge, according to our hospital protocol, 51 patients followed the Home Hospitalization protocol with the assistance of a physiotherapist at home for the first week, 17 patients were transferred to a social health center for recovery.

As postoperative complications, we had an early infection that required debridement surgery and it resolved. We also had a case of femoral nerve paresis that resolved in 6 months. We did not have any case of venous thrombosis and we did not have any case of dislocation until the end of the follow-up.

The clinical evaluation at the end of the follow-up, according to the MD Scale, showed the mean value was 16.454 (SD 0.79472), with a survival at the end of the follow-up of 100% of the placed DMC. The radiographic evaluation at the end of the follow-up showed no subsidence of the femoral stems, there was no osteolysis, no periprosthetic ossification or radiolucency. The average acetabular inclination was 44 ° (range 42 ° - 50 °).

## DISCUSSION

Above all, we are aware of the limitations of our work due to the number of cases we present and the follow-up time of these patients. Nevertheless, we believe that it is important to analyze the results of the use of cemented DMC in prosthetic revision surgery with its possible applications.

Recurrent instability remains a difficult problem after THA revision with a recent study reporting a 35% reluxation rate after THA revision due to instability at 15 years.^1,2^ These patients are usually elderly people who have undergone hip surgery several times and who usually have multiple underlying pathologies associated with it.^4,5^ All this means that they can be considered high-risk patients in revision surgery. In our series, the mean age of revision surgery was 77.39 years (range 46 to 89 years). As a medical history, we have: hip reoperation patients, diabetes mellitus, hypertension, obesity, neuromuscular diseases, heart disease.

The objective of primary hip surgery is to restore the original anatomy and biomechanics of the hip by placing a THA.^17^ For this reason, good preoperative planning is always advised through the use of templates. However, in revision surgery, preoperative planning must be more careful, it must include the evaluation of the soft tissues, especially the abductor muscles, the bone stock and its condition, knowing well the technical possibilities that we have, and above all it is very surgeon's experience is important.^17^

Today we have different resources in revision surgery. Hailer conducted a study of the Swedish Registry of Hip Arthroplasty between 2005 and 2010 and reported 399 revision procedures for THA due to dislocation, being more frequent in the posterior approach than the lateral or anterior approaches.^18^ Another important factor, which we have already mentioned, is the tension of the abductor muscles.^19^ We know that we can modify it by lateralizing the femur with the lateralized ofset stems or with the larger diameter heads.^18^ For this reason, it is advisable to change both prosthetic components at the time of THA revision surgery. But on many occasions it is difficult for the surgeon to change a component that is not loose due to the danger of associated bone fractures. In acetabular bone defects, the placement of jumbo-type acetabular components has been proposed, successfully reported in some studies,^19^ the use of support rings associated with allograft placement,^20,21^ the use of large diameter has helped a lot in revision surgery.^22^ But we know that these heads increase torsional forces at the junction of the trunion and the heads and can cause adverse local alterations, in addition to being an important cause of postoperative pain.^22,23^ Despite all these technical aids, we need something more in revision surgery in elderly patients at risk of dislocation. We agree with Chalmers that surgical options are limited and the use of restricted liners is indicated in THA revision surgery.^16^ Increased polyethylenes were initially used in one area of the rim, but have proven to be insufficient. Constrained polyethylenes later appeared, but they have also caused problems.^24^ We currently have the DMC. They began to be used in the 1970s in primary THA surgery with great success.^8^ Currently we have cemented DMC that allow us different possibilities of use. We can cement them directly to the bone in those cases where there is a good bone stock. We can also cement the DMC into the existing well-fixed metal shell in order to shorten the operative time, and reduce blood loss, bone damage, and overall perioperative morbidity.^25^ And finally, we can cement them to a ring in cases of significant Papronsky type III A and B bone defects, associated if necessary with bone allograft for regeneration of bone defects. In our series, 25 cases were placed with a cemented DMC with a Bursch-Sneider ring associated with allograft placement; in 23 cases a DMC was cemented into the existing well-fixed metal Shell; and in 21 cases it was decided to cement the DMC directly to the bone.

One of the objectives of our work is to assess the clinical situation of the patients after the THA revision intervention with the DMC. There are many studies that recognize a good result in the assessment scales in the follow-up of these patients. Philippot recognizes a clinical improvement from 7.1 preoperatively to 15.8 10 years after the intervention according to the MD scale.^26^ More recently, Lamo-espinosa et al. Report a mean preoperative MD score of 10.31 that goes to 15.61 postoperatively in patients undergoing THA revision using the DMC.^10^ In a series of 36 patients considered high risk and submitted to THA revision, Plummer^27^ reported an improved Harris Hip Score by a mean of 45 points with a final mean of 90. In our series we have gone from a preoperative assessment of 8.34, according to the MD scale, to a result at the end of the follow-up of 15.55 (Table 1). With these results we can affirm that the DMC used in the THA review can reliably improve pain and gait in these patients.

Another objective of our study is to assess re-dislocations in patients with DMC after THA revision surgery. Simian^28^ reported a dislocation rate of 1.4% in patients undergoing THA revision, mainly for aseptic loosening and no history of hip instability, with DMC constructions. In a 994 review THA study for all indications, Wegryzn reported a total dislocation rate of 1.5% and the intraprosthetic dislocation rate was 0.2%.^29^ Several reports indicate up to a 30% dislocation rate of acetabular costrainer liner in operated patients at high risk of recurrent dislocation.^30,31^ DMC constructions and restrained liners have different mechanisms for imparting hip stability. In theory, restricted liners restrict the hip to fit prosthetic design in an attempt to compensate for poor soft tissue. In our series we did not have any case of dislocation after review with the CMD in any of the operated groups.

Another of the possible advantages of the DMC is the possibility of the reduction under closed sky when there is a dislocation of the same. We have already commented previously that in many cases there is a concurrence of several instability factors that facilitate recurrent dislocation. Some of these factors can be unpredictable. This is what happens when the existence of neuromuscular diseases and abductor insufficiency of the hip are associated. Sonohata reported a case of dislocation of a line acetabular costrainer cup that was conservatively resolved without the need for surgical intervention,^32^ but most constrainer liners have a broken mechanism and make conservative reduction impossible. However, DMC can be carried out on many occasions a conservative reduction.^16^ But this is not always possible, as reported by Plummer, who reported two DMC dislocations in a series of 36 patients that required surgical intervention at two years of follow-up.^27^

In patients at high risk of dislocation despite multiple unsuccessful surgical attempts to obtain a stable hip replacement, DMC constructions not only allow greater stability, but also allow the option of treating repeated dislocations with closed reduction and braces in instead of needing urgent surgical intervention as usual. the case in most dislocated constricted liners.

## CONCLUSION

As a conclusion to our work, we can affirm that the use of cemented DMC is a good solution in the replacement of THA, especially in cases of reluxation or risk of dislocation due to personal or technical predisposing factors. The use of these cemented DMC can be directly to the bone, into the existing well-fixed metal Shell, or cemented to a reinforcing ring, depending on the acetabular defect. In any case, we need more casuistry and a longer follow-up time.

## References

[1] Woolson ST, Rahimtoola ZO (1999). Risk factors for dislocation during the first 3 months after primary total hip replacement. J Arthroplasty.

[2] Grigoris P, Grecula MJ, Amstutz HC (1994). Tripolar hip replacement for recurrent prosthetic dislocation. Clin Orthop Relat Res & NA.

[3] Levine BR, Springer BD, Golladay GJ (2020). Highlights of the 2019 American Joint Replacement Registry Annual Report. Arthroplast Today.

[4] Rowan FE, Benjamin B, Pietrak JR, Haddad FS (2018). Prevention of Dislocation After Total Hip Arthroplasty. J Arthroplasty.

[5] Lu Y, Xiaoy H, Xue F (2019). Causes and treatment options for dislocation after total hip replacement. Exp Ther Med.

[6] De Martino I, D’Apolito R, Soranoglou VG, Poultsides LA, Sculco PK, Sculco TP (2017). Dislocation following total hip arthroplasty using dual mobility acetabular components: a systematic review. Bone Joint J.

[7] Vasukutty NL, Middleton RG, Young P, Uzoigwe C, Barkham B, Yusoff S (2014). A double mobility acetabular implant for primary hip arthroplasty in patients at high risk of dislocation. Ann R Coll Surg Engl.

[8] Godoy-Monzona D, Garcia-Mansilla A, Buljubasicha M, Cid-Casteulanib A, Valentinic R, Double mobility system. (2020). The French solution in elderly patients with intracapsular hip fracture and high risk of dislocation Dual mobility system. Rev Esp Cir Ortop Traumatol (Engl Ed).

[9] Kaiser D, Kamath AF, Zingg P, Dora C. (2015). Double mobility cup total hip arthroplasty in patients at high risk for dislocation: a single-center analysis. Arch Orthop Trauma Surg.

[10] Lamo-Espinosa JM, Gómez-Álvarez J, Gatica J, Suárez Á, Moreno V, Díaz de Rada P (2021). Cemented Dual Mobility Cup for Primary Total Hip Arthroplasty in Elder Patients with High-Risk Instability. Geriatrics (Basel).

[11] Dikmen G, Ozden VE, Karaytug K, Tozun R. (2019). Dual-mobility cups in revision acetabular reconstructions: Short-term outcomes in high-risk patients for instability. Acta Orthop Traumatol Turc.

[12] Hailer NP, Weiss RJ, Stark A, Kärrholm J (2012). Dual-mobility cups for revision due to instability are associated with a low rate of re-revisions due to dislocation: 228 patients from the Swedish Hip Arthroplasty Register. Acta Orthop.

[13] Zhang Z, Xu G, Cao L, Sun W, Zeng X, Xiong N (2021). Dual-Mobility Cup Total Hip Arthroplasty for Displaced Femoral Neck Fractures: A Retrospective Study with a Median Follow-Up of 5 Years. Geriatr Orthop Surg Rehabil.

[14] Vasukutty NL, Middleton RG, Matthews EC, Young PS, Uzoigwe CE, Minhas THA (2012). The double-mobility acetabular component in revision total hip replacement: the United Kingdom experience. J Bone Joint Surg Br.

[15] Placella G, Bettinelli G, Pace V, Salini V, Antinolfi P. (2021). Dual mobility for total hip arthroplasty revision surgery: A systematic review and metanalysis. SICOT J.

[16] Chalmers BP, Pallante GD, Taunton MJ, Sierra RJ, Trousdale RT (2018). Can Dislocation of a Constrained Liner Be Salvaged With Dual-mobility Constructs in Revision THA?. Clin Orthop Relat Res.

[17] Faldini C, Stefanini N, Fenga D, Neonakis EM, Perna F, Mazzotti A (2018). How to prevent dislocation after revision total hip arthroplasty: a systematic review of the risk factors and a focus on treatment options. J Orthop Traumatol.

[18] Huten D, Fournier Y, Gicquel T, Bertho P, Basselot F. (2020). Risk factors for dislocation after revision total hip arthroplasty with a dual-mobility cup. Matched case-control study (16 cases vs. 48 controls). Rev Esp Cir Ortop Traum.

[19] Nwankwo CD, Ries MD (2014). Do jumbo cups cause hip center elevation in revision THA? A radiographic evaluation. Clin Orthop Relat Res.

[20] Morales De Cano JJ, Guillamet L, Perez Pons A (2019). Acetabular reconstruction in Paprosky tipe III defects. Acta Ortop Bras.

[21] Odri GA, Padiolleau GB, Gouin FT (2014). Oversized cups as a major risk factor of postoperative pain after total hip arthroplasty. J Arthroplasty.

[22] Weiser MC, Lavernia CJ (2017). Trunnionosis in Total Hip Arthroplasty. J Bone Joint Surg Am.

[23] Malahias MA, Ma QL, Gu A, Ward SE, Alexiades MM, Sculco PK (2020). Outcomes of Acetabular Reconstructions for the Management of Chronic Pelvic Discontinuity A Systematic Review. J Arthroplasty.

[24] Song JH, Kwon WH, Oh SB, Moon KH (2020). Use of a Constrained Acetabular Liner to Prevent and Treat Recurrent Dislocation after Total Hip Replacement Arthroplasty. Orthop Surg.

[25] Wegrzyn J, Saugy CA, Guyen O, Antoniadis A. (2020). Cementation of a Dual Mobility Cup Into an Existing Well-Fixed Metal Shell: A Reliable Option to Manage Wear-Related Recurrent Dislocation in Patients With High Surgical Risk. J Arthroplasty.

[26] Philippot R, Adam P, Farizon F, Fessy MH, Bousquet G. (2006). [Survival of cementless dual mobility sockets: ten-year follow-up]. Rev Chir Orthop Reparatrice Appar Mot.

[27] Plummer DR, Christy JM, Sporer SM, Paprosky WG, Della Valle CJ (2016). Dual-Mobility Articulations for Patients at High Risk for Dislocation. J Arthroplasty.

[28] Simian E, Chatellard R, Druon J, Berhouet J, Rosset P. (2015). Dual mobility cup in revision total hip arthroplasty: dislocation rate and survival after 5 years. Orthop Traumatol Surg Res.

[29] Wegrzyn J, Tebaa E, Jacquel A, Carret JP, Béjui-Hugues J, Pibarot V (2015). Can Dual Mobility Cups prevent Dislocation in All Situations After Revision Total Hip Arthroplasty?. J Arthroplasty.

[30] Chalmers BP, Arsoy D, Sierra RJ, Lewallen DG, Trousdale RT (2016). High Failure Rate of Modular Exchange with a Specific Design of a Constrained Liner in High-Risk Patients Undergoing Revision Total Hip Arthroplasty. Arthroplasty.

[31] Della Valle CJ, Chang D, Sporer S, Berger RA, Rosenberg AG, Paprosky WG (2005). High failure rate of a constrained acetabular liner in revision total hip arthroplasty. J Arthroplasty.

[32] Sonohata M, Waewsawangwong W, Goodman SB (2012). Successful closed reduction of a dislocated constrained total hip arthroplasty: a case report and literature review. Open Orthop J.

